# Plane Wave Diffraction by a Finite Plate with Impedance Boundary Conditions

**DOI:** 10.1371/journal.pone.0092566

**Published:** 2014-04-22

**Authors:** Rab Nawaz, Muhammad Ayub, Akmal Javaid

**Affiliations:** 1 Department of Mathematics, COMSATS Institute of Information Technology, Wah Cantt., Pakistan; 2 Department of Mathematics, Quaid-i-Azam University, Islamabad, Pakistan; University College London, United Kingdom

## Abstract

In this study we have examined a plane wave diffraction problem by a finite plate having different impedance boundaries. The Fourier transforms were used to reduce the governing problem into simultaneous Wiener-Hopf equations which are then solved using the standard Wiener-Hopf procedure. Afterwards the separated and interacted fields were developed asymptotically by using inverse Fourier transform and the modified stationary phase method. Detailed graphical analysis was also made for various physical parameters we were interested in.

## Introduction

Diffraction theory can be applied successfully to reduce the noise due to heavy traffic, environmental pollution and industrial growth by means of barriers in heavily built up areas. A barrier should be good attenuator of sound and inexpensive at the same time. Such barriers may have absorbing lining on the surfaces and satisfy impedance boundary conditions as well. The scattering of sound and electromagnetic waves has been studied extensively since the half plane problems were investigated by Poincare [1] and Sommerfeld [2]. Many classical problems related to electromagnetic waves diffraction due to line source and point source have been studied so far. These problems constitute a canonical problem for the GTD (geometrical theory of diffraction). Scattering analysis by metallic tapes on paneled compact range reflectors [3] and the line source diffraction of electromagnetic waves by a perfectly conducting half plane was investigated by Jones [4]. Rawlins [5] then considered line source diffraction by an acoustically penetrable or an electromagnetically dielectric half plane having smaller width as compared to the incident wave length. In continuation to this, diffraction by an absorbent semi-infinite plane having different impedance faces [6] is also examined. Rawlins [7] used Ingard's conditions [8] on the boundaries to discuss scattering of sound waves by a half plane. Later on Rawlins' idea is extended to calculate the diffraction by finite strip [9] and diffraction of spherical acoustic wave from an absorbing plane [10]. A related study of diffraction by a finite airfoil in uniform flow is presented by Jeon et al. [11]. Myers' presented an improved form of impedance boundary conditions [12] which were used in [13], [14] for sound wave diffraction problems.

Diffraction by strips is a significant and classical subject both in electromagnetic and acoustic wave theory. In specific, scattering from resistive, conductive and impedance strips have been considered by Herman et al. [15], while, Senior has also made an attempt to solve a problem related to resistive strip configuration [16]. Many analytical, numerical or approximate analytical methods have been used to study a single or multiple diffraction patterns from a strip. To name a few for example, geometrical theory of diffraction [17], Kobayashi's potential method [18], [19] spectral iteration technique (SIT) [20], method of successive approximations [21] and the W-H technique [22] have positively been utilized. Some recent advances in the literature are also found on Bessel's potential spaces [23] and Maliuzhinetz-Sommerfeld integral representation [24].

Keeping in view the aforementioned studies, the major aim of this article is to discuss a wave diffraction problem relating field and its normal derivative as first order impedance conditions which are referred to as standard impedance conditions. These conditions are used as they introduce simplification in calculations to make the problem tractable and to achieve a solution simple enough to use. The impedance conditions can be used effectively for the problems dealing with material surfaces whose solution would be impractical without them. Such conditions are widely used to give analytical solutions to canonical problems. This article provides the comprehensive treatment of impedance boundary conditions applied to electromagnetic. The analytical solutions are amenable to develop high frequency electromagnetic scattering codes and should therefore be of interest to practicing engineers as well as researchers concerned with high frequency diffraction by impedance structures. As mentioned earlier, the finite strip problems have been solved by many researchers who considered different impedance boundary conditions. In the present analysis, the solution to plane wave diffraction by a finite conducting plate with impedance type boundary conditions is produced by the two edges of the finite plate.

The structure of the paper is organized as follows. In Section 0, governing problem which is composed of Helmholtz's equation, impedance boundary conditions and continuity conditions, is stated along with its geometrical configuration. The integral transforms are introduced to convert the problem in complex 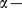
plane so that two unknown functions are defined. The three part boundary value problem is simplified in terms of two Wiener-Hopf functional equations in Section 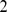 from which we derive integral equations in Section 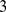 using the standard Wiener-Hopf procedure. The procedure is inspired by the book of Noble [22] which is concerned with the application of the Wiener-Hopf technique to the problems involving semi-infinite and finite geometries and discusses a wide range of extensions. In Section 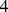, analytic approximation for the two unknown functions are derived using asymptotic analysis of the integral equation for large complex argument. The analytical expressions for separated and interacted fields at both edges are computed. In Section 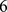 the amplitude of the separated field (which contributes in a physical situation) versus observation angle is tested graphically while problem is concluded in Section 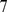 It is mentioned that the time factor is supposed to be 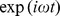 and neglected throughout the analysis.

## Statement of the Boundary Value Problem

This section is dedicated to yield the geometric configuration, governing mathematical equation, corresponding boundary conditions and the transformation used to obtain standard Wiener-Hopf functional equations.

**Figure 1:**
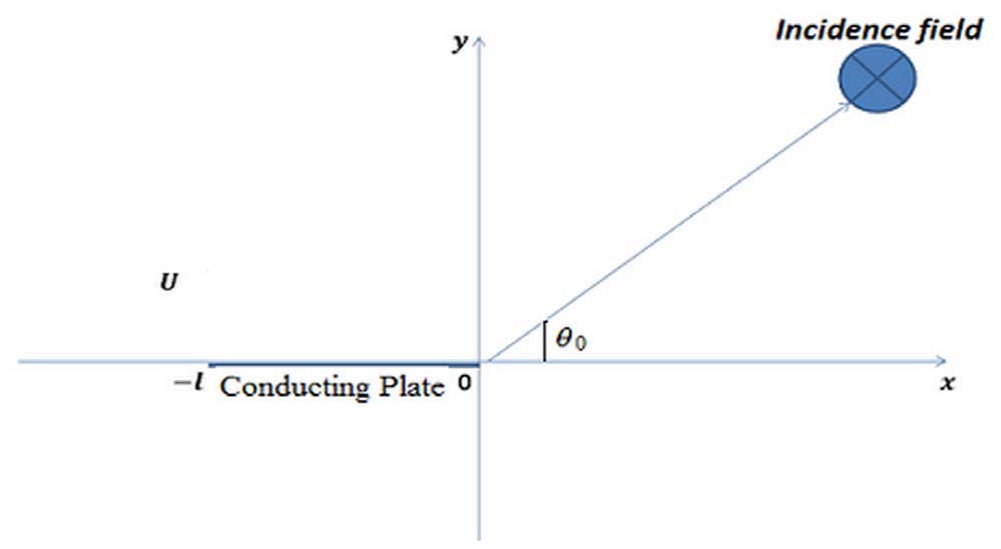
Geometrical configuration of the problem.

Consider the scattering of a time harmonic, plane wave incidence by an impedance finite plate having specific impedance say 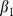. A plate of length 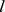 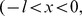 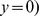 is encountering a small gust with uniform flow 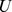 parallel to the finite plate as shown in Figure 1.

The finite plate is assumed to be infinitely thin and straight. For analysis purpose, it is convenient to express the total electric field as follows
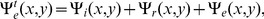
(1)where 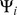 is the plane wave incident field and is given by
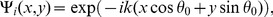
(2)while 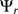 denotes the field reflected from the finite plate at 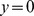 and is given by
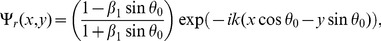
(3)here for an absorbent surface it is required that 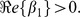 The diffracted electric field 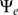 satisfying the following Helmholtz's equation in the range 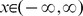 is given by
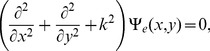
(4)where 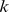 is the free space wave number and for analytic convenience we shall assume that 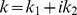 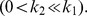 It is supposed that the medium is slightly lossy and the solution for real 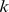 is obtained by letting 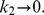 The under considered boundary value problem is expressed in terms of the reduced potential in dimensionalized form, and it is appropriate to denote separated field in the different regions.

As we are interested in determining the diffracted field due to plane wave incidence on the impedance finite plate, Neumann and Dirichlet conditions are imposed along the plate line in mixed type. Different impedance conditions are imposed on upper and lower faces of the conducting plate. Therefore the total diffracted field 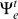 (which may be named as diffracted electric field due to a conducting plate) is to be determined with the following boundary and continuity conditions
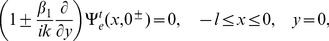
(5)and



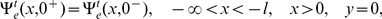
(6)

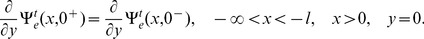
(7)


The boundary conditions (5) are the first order impedance conditions relating field and its normal derivative as outlined by Senior et al. [25]. These impedance boundary conditions were subsequently used to model radio waves propagation along the surface of earth and near conducting obstacles. The detail discussion and practical importance of these impedance conditions can be found in [26]. The obstacle (finite conducting plate) occupies 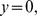 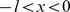 with the velocity of the moving fluid parallel to the 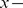
axis having magnitude 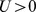. The fluid flow is considered as uniform flow moving along the plate. The governing equations are linearized and the special effects of viscosity, thermal conductivity and gravity are ignored while the fluid is assumed to have a constant density (incompressible fluid) and sound speed 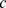.

## Problem in Transform Domain

For the solution of boundary value problem (4–7), let us introduce Fourier transform with respect to variable 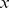 as
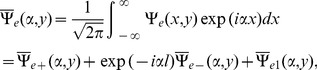
(8)for 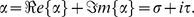 While taking into account the asymptotic behaviors of 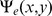 for 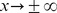 as



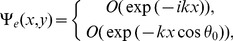
(9)

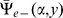
 is a regular function of 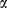 in 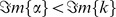 and 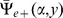 to be regular in 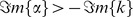 and 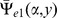 to be analytic in the common region 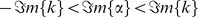 which will provide the analytic region for the use of Wiener-Hopf technique, hence.
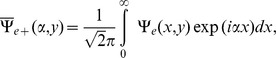





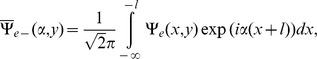





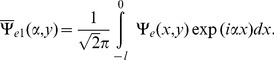
(10)


Now for a plane wave incident on a finite plate, 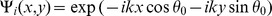 and the incident field in the transformed domain in the region 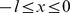, 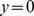 gives.
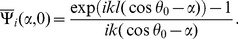
(11)


Also the reflected field 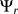 in the transformed domain 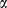 is given by.
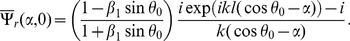
(12)


The Fourier transform of Eqs. (4–7) yields.
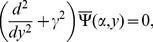
(13)where 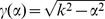 with 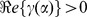, also equation (13) is valid for any 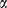 in the strip 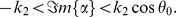 The Fourier transform of boundary conditions (5–7) gives



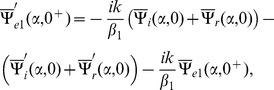
(14)

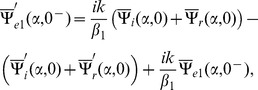
(15)and
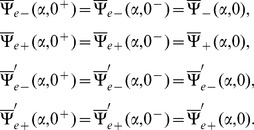
(16)


## Derivation of Wiener-Hopf Equations

The solution of Eq. (13) satisfying radiation condition as 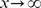 is given by.
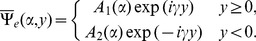
(17)


Now with the help of Eqs. (14–17), the following Wiener-Hopf functional equations are computed
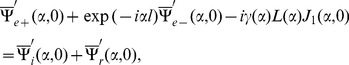
(18)




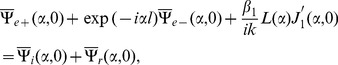
(19)where



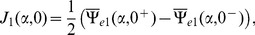
(20)

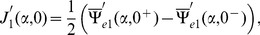
(21)




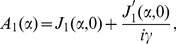
(22)




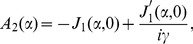
(23)




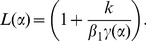
(24)


Since Eqs. (18–19) are the Wiener-Hopf equations therefore we proceed to find the solution for these equations in next section.

## Wiener-Hopf Method

In order to solve the model problem, the intention was, and is, to see the effect of incident wave (which ultimately produces a diffracted field) on a finite conducting plate while considering the impedance boundary conditions. The functional equations (18–19) for the three part boundary value problem are analyzed rigorously using the Wiener-Hopf technique. The main feature of this technique is that it is not fundamentally numerical in nature and thus allows additional insight into the mathematical and physical structure of the diffracted field. The kernel factor appearing in (24) is factorized as
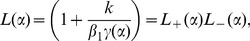
(25)and
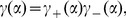
(26)where 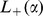 and 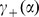 are regular for 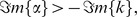 i. e., for upper half plane and 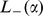 and 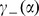 are regular for 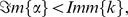 i. e., lower half plane. The factorization of such factors is discussed in [21]. Injecting the value of 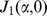 and 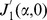 from Eqs. (18–19), into (22) and (23), it is found
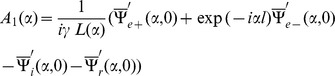





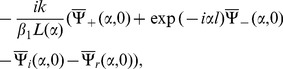
(27)and
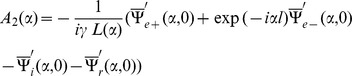





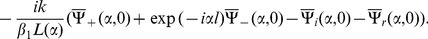
(28)


**Figure 2:**
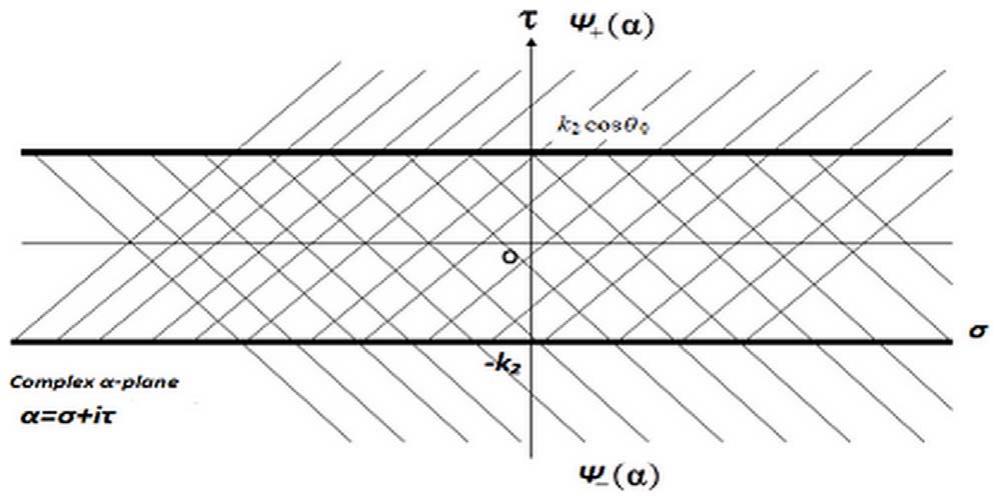
Representation of complex 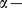
plane.

Making use of Eqs. (11) and (12) in Eqs. (18) and (19) to obtain
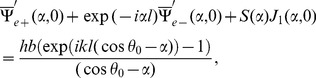
(29)and
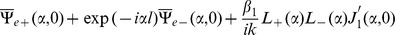





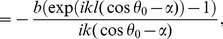
(30)where 
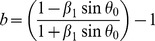
 and 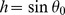 with



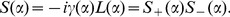
(31)Here 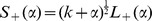 and 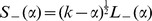 are regular in upper and lower half plane 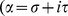, 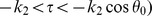, respectively (complex 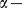
plane is shown in Figure 2.

Fortunately equations of types (29) and (30) have been considered by Noble [22] and an analysis based on the same path, may be utilized to obtain an approximate solution for large 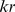 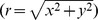. On equating the terms of Eqs. (29–30) with negative sign on one side of the equation and the terms with positive sign on the other side results into a same function say 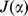. Analytic continuation and extended form of Liouville's theorem extended the function 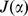 throughout the complex 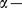
plane so that the entire function 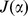 which appears in terms of a polynomial is equated to be zero. Omitting the details of calculations and following the procedure given in [22], it is found that.
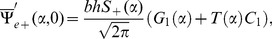
(32)




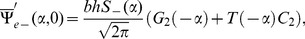
(33)




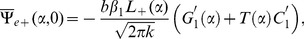
(34)




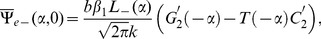
(35)where



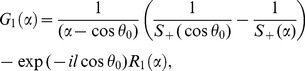
(36)

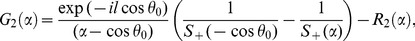
(37)




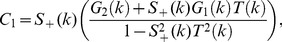
(38)




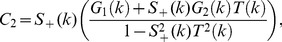
(39)




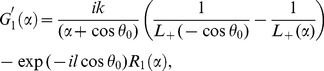
(40)




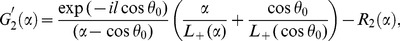
(41)




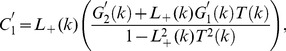
(42)




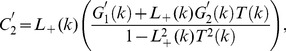
(43)




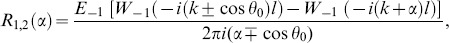
(44)




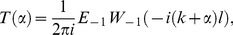
(45)




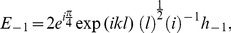
(46)and
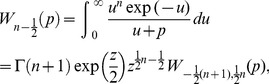
(47)where 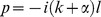 and 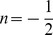. 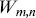 is known as a *Whittaker* function.

Now making use of Eqs. (32–35) in Eqs. (27–28), we get
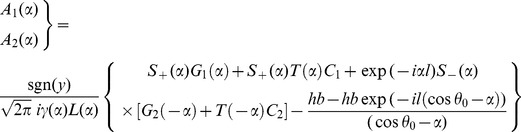





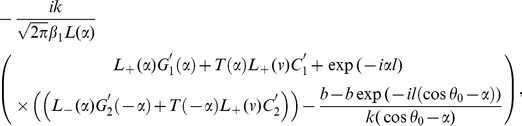
(48)where 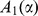 corresponds to 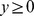 and 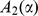 corresponds to 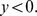 Now, we shall derive a diffracted field expression explicitly in the real space by using the results obtained in (48). The diffracted field 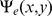 is obtained by taking the inverse Fourier transform of Eq. (17), that is
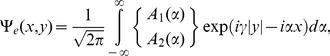
(49)where 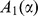 and 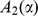 are given in Eq. (48). Substituting the value of 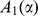 and 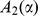 from Eq. (48) into Eq. (49) and using the approximations (36–47), one can break up the field 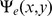 into two parts
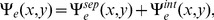
(50)where
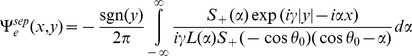





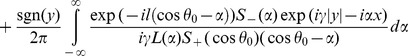





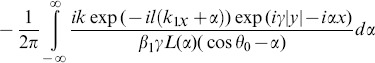





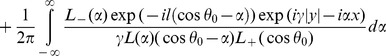





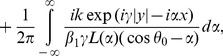
(51)and
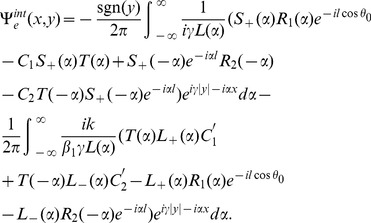
(52)


In above Eqs. (51–52), 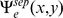 comprises of two parts each representing the diffracted field produced by the edges of conducting plate at 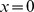 and 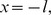 respectively, although the other edges were absent while 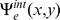 yields the interaction of one edge upon the other.

## Acquisition of Diffracted Field

The diffracted field in the far zone may now be calculated by evaluating the integrals appearing in Eqs. (51–52), asymptotically [27]. For that introducing the polar coordinates as 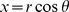, 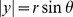 and deforming the contour by the transformation
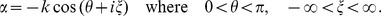



Hence, for large 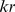, Eqs. (51–52) take the following forms
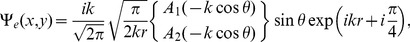
(53)with
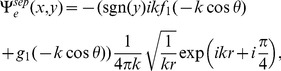
(54)and
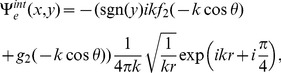
(55)where 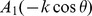 and 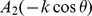 can be found from Eq. (48), while
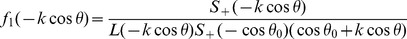





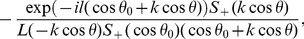
(56)




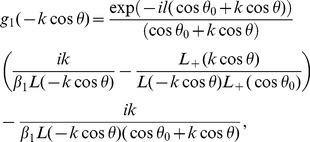
(57)




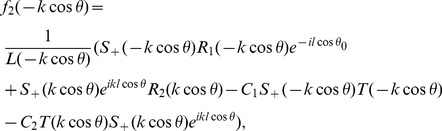
(58)




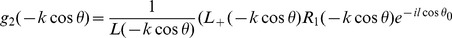


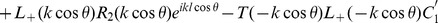





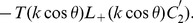
(59)


Eq. (53) gives the far field asymptotic expression of the diffracted field for 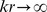 which is also the asymptotic expansion of 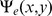 valid for any value of observation angle everywhere in the space. It is noted that the separated field represents the field diffracted by the edges at 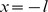 and 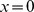 plus the additional contribution to the geometrical wave field not included in the incident field. The separated terms are the resultant wave field which will contribute in the physical insight. Whilst interacted field gives the interaction of one edge with the other but It gives no physical insight. Only the separated field is discussed numerically as it provides the physical insight to the diffraction problem at a defined boundary. Moreover the interaction field is produced due to double diffraction of the two edges which is already considered as separated field is calculated by the edges at 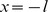 and 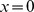. Also the contribution coming from the interacted terms is eliminated when the plate length is widened up to infinity and only the separated terms will result into the diffracted field. Therefore, we only discuss separated field computationally in the subsequent section.

### Numerical Results and Discussion

**Figure 3:**
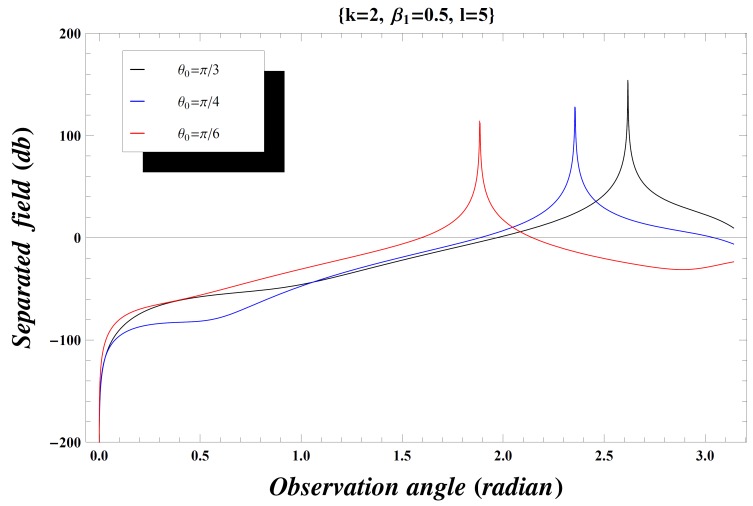
Separated field versus observation angle for different values of incidence angle 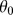 when 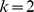.

**Figure 4:**
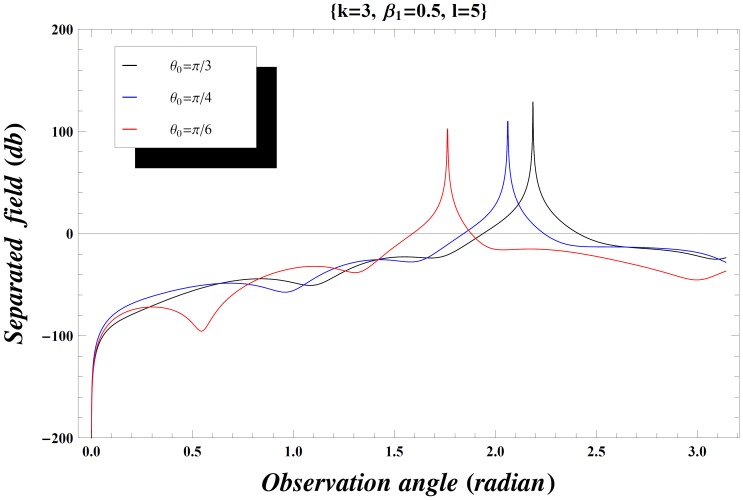
Separated field versus observation angle for different values of incidence angle 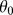 when 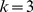.

**Figure 5:**
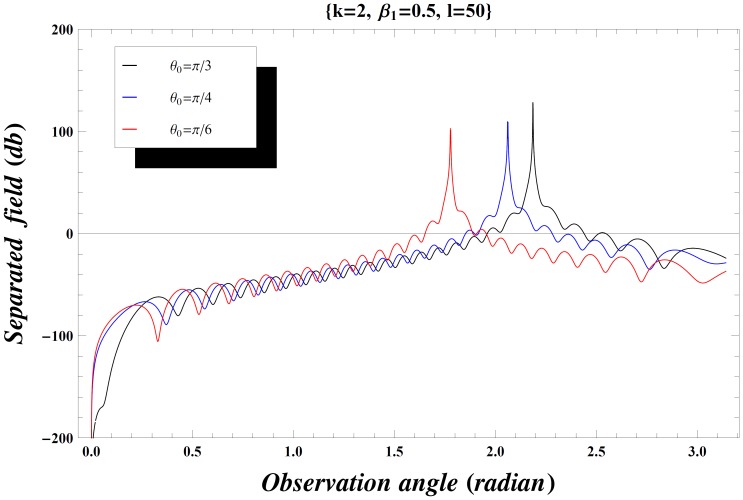
Separated field versus observation angle for different values of incidence angle 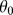 when 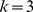 and plate length 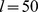.

**Figure 6:**
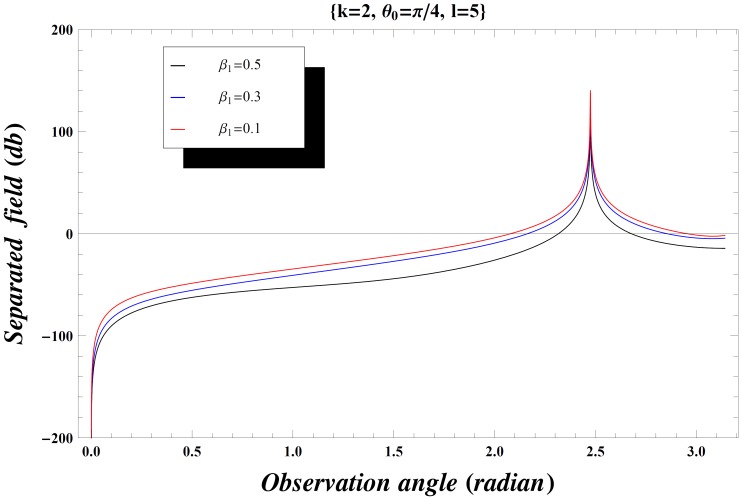
Separated field versus observation angle for different values of admittance parameter 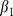 when 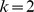.

The effect of the values of incident angle, plate length, specific impedance and the wave number on the diffraction phenomenon is observed computationally by displaying the variation of the separated field (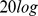) versus observation angle. In Figures 3–4, the results are presented for the different values of incident angle 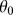 by fixing all other parameters. On increasing the value of incident angle, the amplitude of the separated field is increased and is maximum for the value of incident angle where 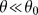. Also while deforming the contour into a hyperbola, we make the transformation.
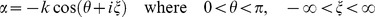
so that the contour over 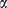 goes into a hyperbola. The two hyperbolae will not cross each other if 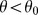 but if the inequality is inverted, there will be a contribution from the pole which actually cancels the incident wave in the shadow region. In Figure 4, the value of wave number is slightly increased which results in little oscillations in the graph while Figure 5 is plotted by extending the plate length. Obviously the overall behavior remains same but the amplitude of the separated field decreases by increasing the plate length and the incident angle. Figures 6–8 are plotted for the different values of specific impedance by fixing all other parameters. It can be seen that by increasing the specific impedance the amplitude of the separated field reduces considerably. It is observed from all the figures that the scattered far field has maximum peaks at different values of observation angle between 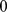 and 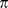. In Figures 9–11, the amplitude of the separated field versus observation angle is observed for different values of wave number 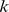. The separated field increases by increasing the value of wave number, *i. e.*, the wave dimension then moves towards the high frequency range.

**Figure 7 pone-0092566-g007:**
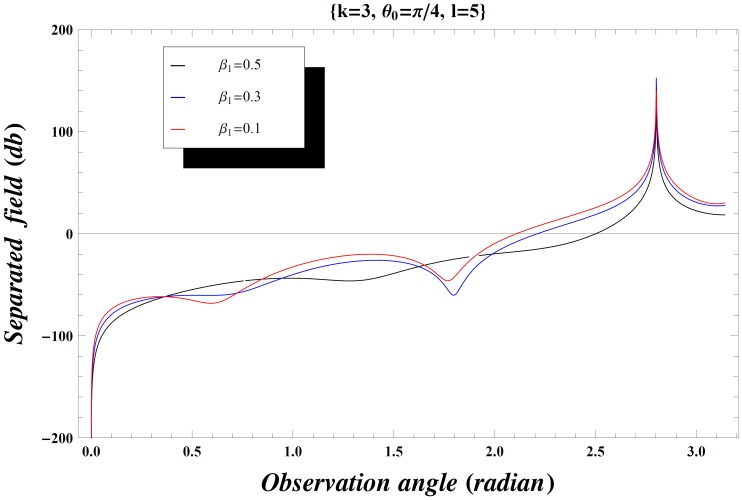
Separated field versus observation angle for different values of admittance parameter 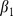 when 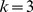.

**Figure 8:**
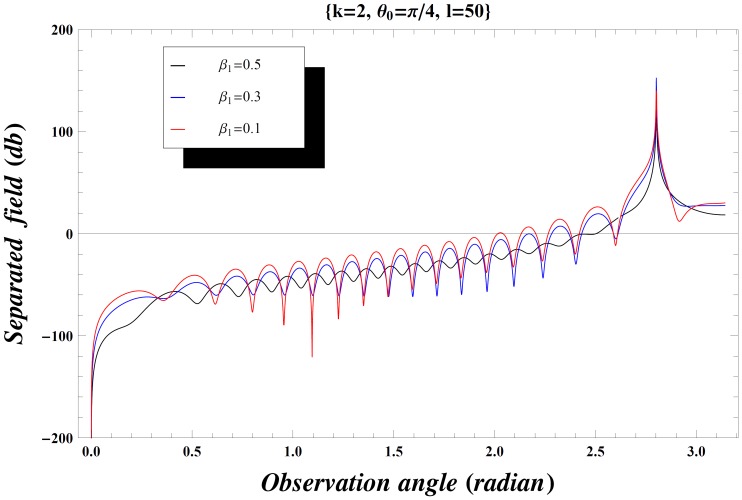
Separated field versus observation angle for different values of admittance parameter 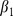 when 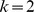 and plate length 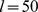.

**Figure 9:**
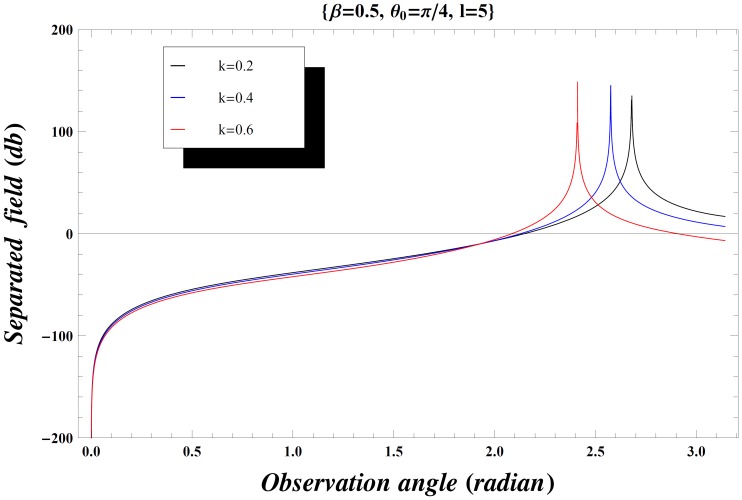
Separated field versus observation angle for different values of wave number 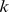 when 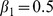.

**Figure 10 pone-0092566-g010:**
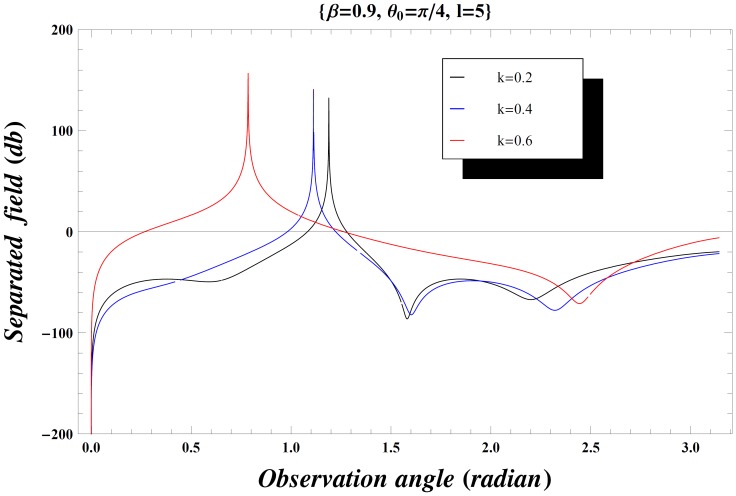
Separated field versus observation angle for different values of wave number 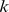 when 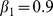.

**Figure 11:**
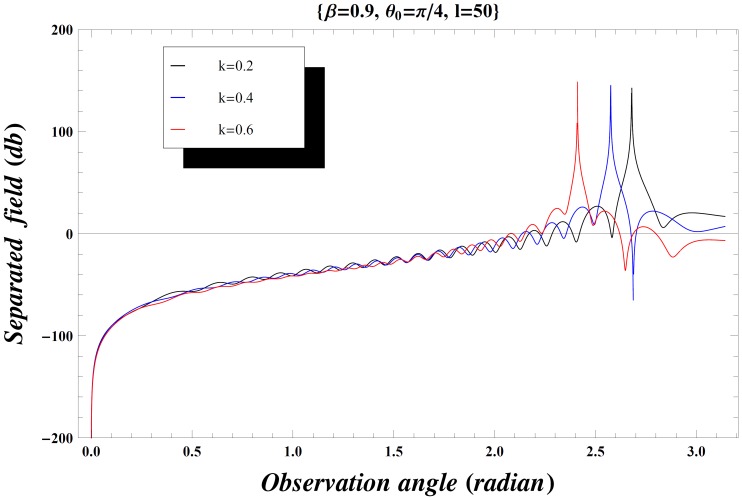
Separated field versus observation angle for different values of wave number 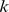 when 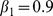 and the plate length 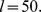

**Figure 12:**
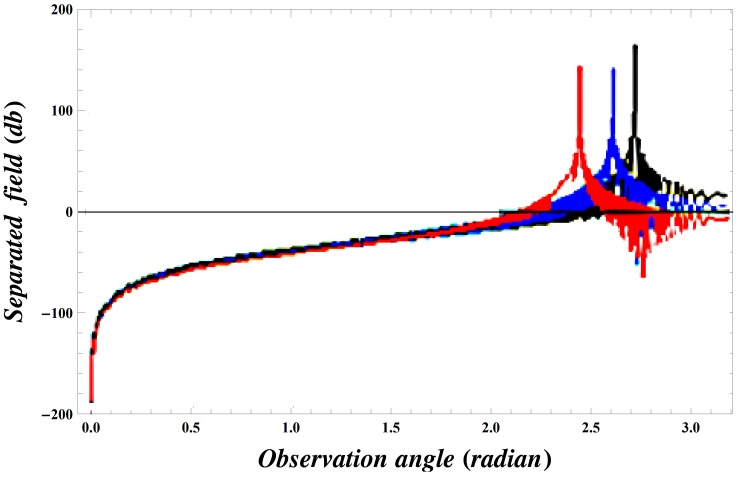
Separated field versus observation angle for values of 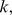 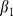 and 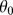 with plate length 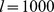.

It is interesting to note that, while increasing the length of plate, the scattered far-field intensity has sharp peaks for almost every value of observation angle. If we keep on increasing this length, the graphs for all the values of 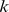, 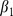 and 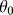 will behave like Figure 12. That is, the overall behavior for different parameters remains alike but the intensity of separated field got sharp oscillating peaks and possibly this could be the results for the half plane. Consideration on the structure of a finite conducting plate may offer a physical understanding of the scattering phenomenon at these particular values of observation angle.

## Concluding Remarks

The principle concern of this investigation is to discuss plane wave diffraction by a finite conducting plate with different impedance boundary conditions. The two edges of the finite plate give rise to two diffracted fields (one from each edge), that is, the separated field and the interacted field (due to interaction of one edge upon the other). Explicit expressions for the separated and the interacted terms are obtained asymptotically using the modified stationary phase method. The final solution obtained here is rigorous and uniformly valid for impedance boundaries. According to Rawlins [28] the impedance half plane gives better attenuation results than a completely rigid semi-infinite plane for singly diffracted fields. Therefore the impedance finite strip (plate) gives better attenuation results for the separated and interacted fields as compared to a completely rigid strip. As the mod value of given field is directly proportional to the perturbation sound pressure which ultimately yields a measure of sound intensity, few graphs showing the effects of various parameters on the separated diffracted field are presented and discoursed. The consideration of plane wave diffraction by finite plate will go a step further to complete the discussion for line source/point source and slit/half plane. If the obstacle (whether finite or semi-infinite) is taken as a noise barrier, the consideration of trailing edge situation can also be more efficient to reduce the noise in this region.
